# Evaluating the Quality, Readability, and Activity of Online Information on Brain Arteriovenous Malformations

**DOI:** 10.7759/cureus.45984

**Published:** 2023-09-26

**Authors:** Mehul Mehra, Pierce A Brody, Sai Suraj Kollapaneni, Om Sakhalkar, Scott Rahimi

**Affiliations:** 1 Neurological Surgery, Medical College of Georgia, Augusta University, Augusta, USA

**Keywords:** hereditary hemorrhagic telangiectasias, cerebral aneurysms, arteriovenous malformations, readability, quality of information, seasonality, google trends

## Abstract

Introduction

Brain arteriovenous malformations (AVMs) are vascular deformities created by improper connections between arteries and veins, most commonly in the brain and spinal cord. The management is complex and patient-dependent; further understanding of patient education activities is imperative. Internet access has become more ubiquitous, allowing patients to utilize a large database of medical information online. Using Google Trends (GT) (Google LLC, Mountain View, CA, USA), one can see the public interest in a particular topic over time. Further, when presented with numerous search results, patients may not be able to identify the highest-yielding resources, making objective measures of information quality and readability imperative.

Methods

A GT analysis was conducted for “hereditary hemorrhagic telangiectasia,” “cerebral aneurysm,” and “arteriovenous malformation”. These relative search volumes (RSV) were compared with the 2017 to 2019 annual USA AVM diagnosis quantity for correlation. These RSVs were also compared with the 2017 to 2019 annual USA deaths due to cerebral hemorrhagic conditions. One search was conducted for “brain arteriovenous malformation”. Since most users looking for health information online use only the first page of sources, the quality and readability analyses were limited to the first page of results on Google search. Five quality tools and six readability formulas were used.

Results

Pearson’s correlation coefficients showed positive correlations between USA AVM RSVs and annual AVM deaths per capita from 2017 to 2019 (R^2^=0.932). The AVM annual diagnosis quantity and AVM RSVs showed a strong positive correlation as well (R^2^=0.998). Hereditary hemorrhagic telangiectasia and cerebral aneurysms had strong positive correlations between their RSVs and their corresponding annual diagnoses in the 2017 to 2019 time period (R^2^=0.982, R^2^=0.709). One-way ANOVA, for USA’s 2004 to 2021 AVM RSVs and 2004 to 2019 deaths per capita, displayed no month-specific statistically significant repeating pattern (all p>0.483). The DISCERN tool had four websites that qualified as “poor” and five as “good.” The average score for the tool was “good.” The Journal of the American Medical Association (JAMA) benchmark scores were very low on average, as four websites achieved zero points. There was a wide variance in the currency, relevance, authority, accuracy, and purpose (CRAAP) scores, indicating an inconsistent level of webpage reliability across results. The patient education materials assessment tool (PEMAT) understandability (86.6%) showed much higher scores than the PEMAT actionability (54.6%). No readability score averaged at or below the American Medical Association (AMA)-recommended sixth-grade reading level.

Conclusion

These GT correlations may be due to patients and families with new diagnoses researching those same conditions online. The seasonality results reflect that no prior research has detected seasonality for AVM diagnosis or presentation. The quality study showed a wide variance in website ethics, treatment information quality, website/author qualifications, and actionable next steps regarding AVMs. Overall, this study showed that patients are routinely attempting to access information regarding these intracranial conditions, but the information available, specifically regarding AVMs, is not routinely reliable and the reading level required to understand them is too high.

## Introduction

Brain arteriovenous malformations (AVMs) are vascular deformities created by improper connections between arteries and veins, most commonly in the brain and spinal cord, that contribute to 1% to 2% of all strokes [1]. Although AVMs can be sporadic, certain diseases can predispose individuals to their formation, or they can occur concurrently with other anatomic abnormalities. For example, hereditary hemorrhagic telangiectasia (HHT) is a genetic disorder that leads to the improper development of blood vessels and a subsequently higher prevalence of AVMs [2].

A deeper understanding of patient education activities is imperative, as current AVM management is complex and patient-dependent. With modern internet access becoming more ubiquitous, patients now utilize a large database of medical information available online. Patients can self-educate about diseases using a variety of online resources via search engines. Daily, 8.5 billion searches occur on Google Search (Google LLC, Mountain View, CA, USA), making it the most frequently used search engine. Google Trends (GT), a database created by Google, contains the number of search queries about individual topics processed by Google Search since 2004 and normalizes those values against similar topics [3]. Using GT, one can see the public interest in a particular topic over time.

Previous studies have implemented GT data to monitor public interest in certain medical topics. Studies have demonstrated public interest trends across seasonal, demographic, and geographic variations [4]. Monitoring GT data has also been used to supplement current data sources to track contemporary public health issues, as one study used GT data to monitor the opioid overdose epidemic [5]. Furthermore, current and future applications include the ability to predict patient decisions and trends, which can be utilized to make decisions that affect public health [6]. We hypothesize that there will be a statistically significant relationship between the number of Google searches for AVMs and the incidence of brain AVMs.

Secondly, we plan to investigate the quality and readability of the information generated by search queries related to AVMs. The algorithm incorporated into Google Search attempted to present resources deemed relevant to a user’s query. These resources each have unique characteristics, such as origin (i.e., government organization, personal blog), intended purpose (i.e., commercial, educational), and overall quality. When presented with numerous search results, patients may not be able to identify the highest-quality resource. Analysis of online resources using objective rating tools will allow us to evaluate the quality of the online medical information currently available on AVMs. Prior studies investigating other medical topics have concluded that the information quality and readability on websites aimed at educating the public are unreliable [7-9].

Additionally, effective media literacy empowers patients to analyze a wide swath of health information and make informed decisions. Currently, the American Medical Association (AMA) and the National Institute of Health (NIH) recommend that healthcare information should be below a sixth-grade reading level to efficiently inform the public [10]. While information synthesis falls to the patients and their families, providing readable, accurate, and unbiased medical sources for the wider population with varying levels of literacy is the responsibility of the wider healthcare establishment.

The overall intention of this study is to provide an end-to-end analysis of AVM information and activity online. The study comprises an analysis of online activity regarding AVMs as well as an objective analysis of information quality and readability in order to understand the role of the internet in mapping out interests and in the general population’s attempt to self-educate.

## Materials and methods

Google Trends

Due to the status of the data used as public and deidentified, this study was exempt from Institutional Review Board review. The GT data is measured in relative search volumes (RSV). These scores range from 0 to 100 and are a normalized proportion of the absolute search volume compared to the total search volume for the given topics, time periods, and locations specified. The database includes spelling mistakes and plural forms of searches in the analysis of a specified term.

The GT analysis was conducted for “hereditary hemorrhagic telangiectasia,” “cerebral aneurysm,” and “arteriovenous malformation”. This analysis utilized the “health” topic filter using the regions “worldwide” and “USA.” These RSVs were compared with the 2017-2019 Healthcare Cost and Utilization Project Database (HCUP) National Inpatient Sample (NIS) for correlation analysis between RSVs and nationwide diagnoses quantity [11]. These GT scores were also compared with the 2017-2019 CDC Wonder Underlying Cause of Death in the USA for correlation analysis between RSVs and nationwide deaths [12]. The International Classification of Diseases, 10th Revision (ICD-10) codes I67.1 (cerebral aneurysm, nonruptured), I78.0 (HHT), and Q28.2 (AVMs of cerebral vessels) were from CDC Wonder. The correlation analysis was performed with Pearson’s correlation coefficient.

Seasonality was assessed for the GT AVM RSVs from 2004 to 2021 and the CDC Wonder nationwide deaths to determine if specific months had an increase or decrease in public interest, as fluctuations in term interest may indicate increases or decreases in diagnoses and deaths. This part of the analysis was performed by a one-way ANOVA with α = 0.05. Finally, two-tailed, two-sample z-tests compared the effect of the COVID-19 era on RSV trends.

Quality of Internet information

One search was conducted for “brain arteriovenous malformation”, which was the term selected to limit overlap with information regarding the less common AVMs occurring outside of the central nervous system. Since most searchers looking for health information online use only the first page of sources, the quality and readability analyses were limited to the first page presented on Google Search [13]. As the tools selected for the analysis in this study are built for websites only, search results that were labeled as or consisted of only PDFs, videos, advertisements, or duplicate webpages were excluded. Google search was conducted in incognito mode on December 15th, 2021. The search was performed in Augusta, Georgia, in order to obtain results specific to the United States. Two authors were chosen to be co-raters for the selected websites and conducted independent scoring between December 15, 2021, and February 12, 2022. The scoring systems used were the DISCERN instrument, the Journal of the American Medical Association (JAMA) benchmarks, the Health On The Net Foundation code (HONcode), the Patient Education Materials Assessment Tool (PEMAT), and the currency, relevance, authority, accuracy, and purpose (CRAAP) test.

The DISCERN instrument is a tool for grading health information online, consisting of 16 questions with scores from 1 to 5. A score of 1 is given for a pure 'no', 5 for a pure 'yes', and 2 to 4 are given for websites that only partially fulfill the question. The tool is grouped into three question categories. (1) Is the publication reliable? (2) How good is the quality of information on treatment choices? And (3), what is the overall rating of the publication? Furthermore, websites were given one of the five grades: excellent (63-75), good (51-62), fair (39-50), poor (27-38), and very poor (15-26) based on overall scores [14].

The HONcode is an ethical code created by the HON Foundation and endorsed by the World Health Organization. The code is a framework for interpreting health information websites’ ethics. It consists of eight values: authority, complementarity, confidentiality, attribution, justifiability, transparency, financial disclosure, and advertising policy [15]. The JAMA benchmarks are a simplified code of ethics (authorship, attribution, disclosure, and currency). The code is intended to provide users with an efficient tool for judging a website’s ethics [16].

The CRAAP test used in this study is an altered version of a tool that was originally designed to evaluate reliability across many media formats and subjects. This altered CRAAP test was revamped for quality by other researchers for quality of health information analysis. The tool consists of 23 questions for assessing accuracy, authorship, and various biases [17].

The PEMAT is a two-part tool intended to evaluate patient education materials in various media. The questions are split into an understandability section, designed to assess if consumers of various backgrounds can understand the key concepts, and an actionability section, designed to assess how much consumers of various backgrounds are able to do with the information and tools provided [18]. Previous studies have used a nonempirical cutoff of 70%, where scores below would be considered poorly actionable and poorly understandable [19,20].

The HONcode and JAMA benchmarks are designed to evaluate a single linked webpage in the context of the full website. This requires raters to navigate to links within the home website to collect ancillary information. Contrarily, DISCERN, PEMAT, and CRAAP are used to analyze a single webpage or information material only. The PEMAT is the only tool designed to assess media formats other than text, specifically visual aids and actionable tools (e.g., checklists).

The analysis of websites was done with descriptive statistics, as the sample size used was too small for more complex statistics. The inter-rater reliability was analyzed using Cohen’s Kappa test for the rating tool on each website. Cohen’s Kappa results are interpreted as excellent (1.00-0.75), good (0.74-0.60), fair (0.59-0.40), and poor agreement (<0.40). After the inter-rater reliability was calculated, raters discussed the discrepancies and created a set of consensus scores for each website.

Quality analysis of websites was performed with descriptive statistics from the consensus scores, as the sample size used was too small for more complex statistics. Descriptive statistics were further stratified by multiple website characteristics. The first set of groupings were the website categories: government, commercial, and academic/nonprofit/healthcare providers. The second set of groupings was whether websites displayed the HON logo or not. The HON Foundation allows webpages to display the logo if they apply and satisfy the ethical principles set forth by the foundation.

Readability analysis

The websites assessed for quality were also analyzed using readability formulas. The analysis was conducted by pasting only a website’s article text into the Readble calculator (AddedBytes, Horsham, UK) [21]. Descriptive statistics were used again for the readability analysis and were also subdivided by website category and HONcode logo status.

The analysis used six unique formulas to achieve a wider breadth of information. The tests each use a unique set of sentence, word, and overall passage characteristics to output a result communicating the minimum grade level of education required to understand the passage of text. Five of the tests output a grade level: Flesch-Kincaid Grade Level (FKGL), Gunning Fog Index (GFI), Coleman-Liau Index (CLI), Simple Measure of Gobbledygook Index (SMOG), and Automated Readability Index (ARI). The sixth test, Flesch-Kincaid Reading Ease (FKRE), outputs a result that requires further interpretation to determine the grade level.

The FKRE and FKGL each use average sentence length by both the number of words and by syllables per word [22,23]. The GFI examines the proportion of words consisting of three or more syllables relative to the total number of words, along with the average sentence length [24]. The CLI makes use of the average character count per 100 words as well as the average sentence count per 100 words [25]. The SMOG analyzes 10-sentence samples from the beginning, middle, and end of the text, calculating the square root of the quantity of polysyllabic words per sentence [26]. The ARI analyzes the average words per sentence and characters per word [27].

## Results

Google Trends

**Figure 1:**
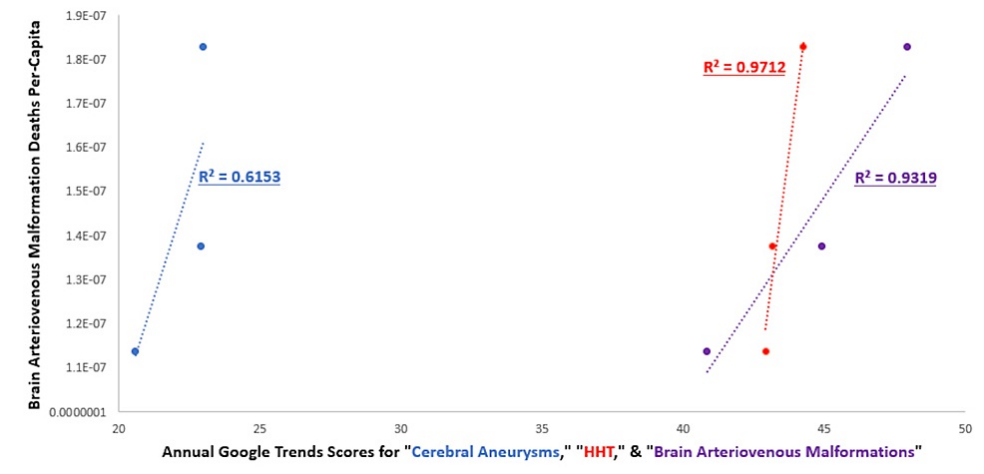
Associations of brain AVM deaths per capita and Google Trends scores with the prevalence of cerebral aneurysms, HHT, and brain AVM AVM: Arteriovenous malformations, HHT: Hereditary hemorrhagic telangiectasias

**Figure 2:**
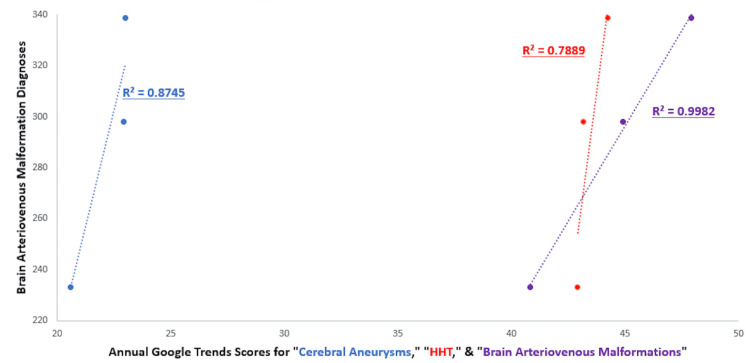
Associations of brain diagnoses and Google Trends scores with the prevalence of cerebral aneurysms, HHT, and brain AVM AVM: Arteriovenous malformations, HHT: Hereditary hemorrhagic telangiectasias

Pearson’s correlation coefficients showed positive correlations between USA AVM RSVs and annual AVM deaths per capita from 2017 to 2019 (R2=0.932) (Figure 1). The AVM NIS annual diagnosis quantity and AVM RSVs showed a strong positive correlation as well (R2=0.998) (Figure 2). The HHT and cerebral aneurysms had strong positive correlations between their RSVs and their corresponding NIS annual diagnoses in the 2017 to 2019 time period (R2=0.982, R2=0.709). The HHT RSVs had a strong positive correlation with CDC Wonder AVM deaths (R2=0.971) and deaths per capita (R2 = 0.971). Cerebral aneurysms exhibited a moderately positive correlation with AVM deaths (R2=0.617) and deaths per capita (R2=0.615). The HHT and cerebral aneurysms also had strong positive correlations between their RSVs and AVM diagnoses in the 2017 to 2019 time period (R2=0.789, R2=0.875).

One-way ANOVA (α = 0.05) for the USA’s 2004 to 2019 (pre-COVID-19) and 2004 to 2021 (with COVID-19) RSVs and 2004 to 2019 deaths per capita displayed no month-specific statistically significant repeating pattern or seasonality (all p > 0.483). November-specific seasonality was detected for worldwide 2004 to 2019 and 2004 to 2021 GT scores (p=0.0189, p=0.0131). Two-tailed, two-sample z-tests evaluated the worldwide and USA average monthly popularity z-scores change between 2004 and 2019 and 2004 and 2021, revealing no statistically significant impact of COVID-19 on RSVs.

Quality of information

The search resulted in nine links without any results meeting the exclusion criteria. There were eight academic/nonprofit/healthcare provider websites, one government website, and zero commercial websites. The single government website (https://medlineplus.gov/ency/article/000779.htm) had better quality scores on every tool except PEMAT understandability when compared to the average academic/nonprofit/healthcare scores (Table 1). There were only two websites that displayed the HONcode logo. When compared to their counterparts without the logo, websites with the logo averaged higher scores in CRAAP, HONcode, JAMA Benchmarks, and PEMATunderstandability while averaging lower scores in DISCERN and PEMAT actionability. Cohen’s Kappa scores averaged excellent inter-rater reliability for each tool individually (Table 2). One website had a Cohen’s Kappa score that qualified as good for DISCERN (https://www.mayoclinic.org/diseases-conditions/brain-avm/symptoms-causes/syc-20350260). All other individual scores qualified as excellent.

**Table 1:** “Brain arteriovenous malformations” Google Search website quality, treatment, and readability scores and statistics for 2021, overall, and by website categories JAMA: Journal of the American Medical Association; PEMAT: Patient Education Materials Assessment Tool; CRAAP: Currency, relevance, authority, accuracy, and purpose; SMOG: Simple Measure of Gobbledygook Index; HONcode: Health On The Net Foundation code

All websites	DISCERN	CRAAP	HONcode	JAMA benchmarks	PEMAT understandability	PEMAT actionability	Flesch-Kincaid Reading Ease Score	Flesch-Kincaid Grade Level	Gunning-Fog Index	Coleman-Liau Index	SMOG Index	Automated Readability Index
Mean	41.6	23.9	3.44	1	86.6%	54.6%	56.0	8.46	10.6	11.0	11.2	8.20
Standard Deviation	4.80	6.39	1.24	1	6.28%	34.4%	9.58	1.87	2.19	1.61	1.56	2.00
Range	36 to 49	15 to 30	2 to 5	0 to 2	75% to 93.8%	0% to 100%	N/A	N/A	N/A	N/A	N/A	N/A
Mean Percentage of Maximum	51.9%	70.3%	43.1%	25.0%	N/A	N/A	N/A	N/A	N/A	N/A	N/A	N/A
Government websites	DISCERN	CRAAP	HONcode	JAMA benchmarks	PEMAT understandability	PEMAT actionability	Flesch-Kincaid Reading Ease Score	Flesch-Kincaid Grade Level	Gunning-Fog Index	Coleman-Liau Index	SMOG Index	Automated Readability Index
Mean	49	30	4	2	85.7%	75.0%	63.8	6.30	7.70	9.30	9.20	5.60
Standard Deviation	N/A	N/A	N/A	N/A	N/A	N/A	N/A	N/A	N/A	N/A	N/A	N/A
Range	N/A	N/A	N/A	N/A	N/A	N/A	N/A	N/A	N/A	N/A	N/A	N/A
Mean Percentage of Maximum	61.3%	88.2%	50.0%	50.0%	N/A	N/A	N/A	N/A	N/A	N/A	N/A	N/A
Academic websites	DISCERN	CRAAP	HONcode	JAMA benchmarks	PEMAT understandability	PEMAT actionability	Flesch-Kincaid Reading Ease Score	Flesch-Kincaid Grade Level	Gunning-Fog Index	Coleman-Liau Index	SMOG Index	Automated Readability Index
Mean	40.6	23.1	3.38	0.875	86.7%	52.0%	55.0	8.73	11	11.2	11.4	8.56
Standard Deviation	4.17	6.38	1.30	0.991	6.70%	35.8%	9.75	1.80	2.03	1.58	1.48	1.87
Range	36 to 46	15 to 29	2 to 5	0 to 2	75% to 93.8%	0% to 100%	N/A	N/A	N/A	N/A	N/A	N/A
Mean Percentage of Maximum	50.8%	68.0%	42.2%	21.9%								
Commercial websites	DISCERN	CRAAP	HONcode	JAMA benchmarks	PEMAT understandability	PEMAT actionability	Flesch-Kincaid Reading Ease Score	Flesch-Kincaid Grade Level	Gunning-Fog Index	Coleman-Liau Index	SMOG Index	Automated Readability Index
Mean	N/A	N/A	N/A	N/A	N/A	N/A	N/A	N/A	N/A	N/A	N/A	N/A
Standard Deviation	N/A	N/A	N/A	N/A	N/A	N/A	N/A	N/A	N/A	N/A	N/A	N/A
Range	N/A	N/A	N/A	N/A	N/A	N/A	N/A	N/A	N/A	N/A	N/A	N/A
Mean Percentage of Maximum	N/A	N/A	N/A	N/A	N/A	N/A	N/A	N/A	N/A	N/A	N/A	N/A
Websites without HONcode	DISCERN	CRAAP	HONcode	JAMA benchmarks	PEMAT understandability	PEMAT actionability	Flesch-Kincaid Reading Ease Score	Flesch-Kincaid Grade Level	Gunning-Fog Index	Coleman-Liau Index	SMOG Index	Automated Readability Index
Mean	43	22.4	3	0.714	84.5%	56.0%	54.7	8.76	11.0	11.0	11.5	8.39
Standard Deviation	4.43	6.58	1	0.951	5.52%	39.6%	10.5	2.04	2.36	1.84	1.65	2.27
Range	37 to 49	15 to 30	2 to 4	0 to 2	75.0% to 93.8%	0% to 100%	N/A	N/A	N/A	N/A	N/A	N/A
Mean Percentage of Maximum	53.8%	66.0%	37.5%	17.9%	N/A	N/A	N/A	N/A	N/A	N/A	N/A	N/A
Websites with HONcode	DISCERN	CRAAP	HONcode	JAMA benchmarks	PEMAT understandability	PEMAT actionability	Flesch-Kincaid Reading Ease Score	Flesch-Kincaid Grade Level	Gunning-Fog Index	Coleman-Liau Index	SMOG Index	Automated Readability Index
Mean	36.5	29	5	2	93.8%	50.0%	60.6	7.4	9.3	10.9	10.1	7.55
Standard Deviation	0.71	0	0	0	0%	0%	4.67	0.00	0.566	0.566	0.354	0.212
Range	36 to 37	29 to 29	5 to 5	2 to 2	93.8% to 93.8%	50% to 50%	N/A	N/A	N/A	N/A	N/A	N/A
Mean Percentage of Maximum	45.6%	85.3%	62.5%	50.0%	N/A	N/A	N/A	N/A	N/A	N/A	N/A	N/A

**Table 2:** Cohen's Kappa Inter-rater reliability overall and by website for 2021 Google Search results for “brain arteriovenous malformations” JAMA: Journal of the American Medical Association; PEMAT: Patient Education Materials Assessment Tool; CRAAP: Currency, relevance, authority, accuracy, and purpose; HONcode: Health On The Net Foundation code

Website number	Website URL	DISCERN	CRAAP	HONcode	JAMA benchmarks	PEMAT understandability	PEMAT Actionability
1	https://www.mayoclinic.org/diseases-conditions/brain-avm/symptoms-causes/syc20350260#:~:text=A%20brain%20arteriovenous%20malformation%20(AVM,AVM%20disrupts%20this%20vital%20process.	0.747	1	1	1	1	1
2	https://www.mayoclinic.org/diseases-conditions/arteriovenous-malformation/symptoms-causes/syc-20350544	1	1	1	1	1	1
3	https://www.stroke.org/en/about-stroke/types-of-stroke/hemorrhagic-strokes-bleeds/what-is-an-arteriovenous-malformation	0.83	1	1	1	1	1
4	https://www.hopkinsmedicine.org/health/conditions-and-diseases/arteriovenous-malformations	0.83	1	1	1	1	1
5	https://www.uclahealth.org/radiology/interventional-neuroradiology/arteriovenous-malformation	1	1	1	1	1	1
6	https://my.clevelandclinic.org/health/diseases/16755-arteriovenous-malformation-avm	0.83	0.788	1	1	1	1
7	https://www.aans.org/Patients/Neurosurgical-Conditions-and-Treatments/Arteriovenous-Malformations	1	1	1	1	1	1
8	https://medlineplus.gov/ency/article/000779.htm	0.909	1	1	1	1	1
9	https://www.bcm.edu/healthcare/specialties/neurosurgery/cerebrovascular-and-stroke-surgery/brain-arteriovenous-malformations-avms	1	1	1	1	1	1
All	All	0.889	0.977	1	1	1	1

Readability

No readability score averaged at or below the recommended sixth-grade reading level (as seen above in Table 1). Of the nine websites, each with six readability scores, there were only three scores at or below a sixth-grade level. The single government website averaged scores translating to lower grade levels in four (FKGL, GFI, SMOG, and ARI) of the six tools. Websites neither with nor without the HONcode logo averaged readability scores at or below a sixth-grade level. Websites with the logo averaged better readability scores in all six of the formulas.

## Discussion

The aim of this study was to analyze the online public interest trends, quality of information, and readability of information on AVMs. The public interest portion of this study showed that there is a strong correlation between AVM online interest and AVM deaths and AVM diagnoses. There was also a strong correlation between the similar neurologic conditions HHT and cerebral aneurysm and their respective annual GT RSVs and diagnosis quantity. This correlation may be due to the fact that patients and families facing new diagnoses are researching those same conditions online. Prior research has shown that a new medical diagnosis leads to a high likelihood of subsequent patient-conducted research online [28]. Furthermore, these same patients also have a higher likelihood of active engagement with their physicians regarding their own knowledge base and treatments [28]. This strong correlation is important, as it is likely that as diagnosis quantities increase, the new AVM patients are increasing their own self-education. However, this analysis is unable to detect causality within the relationship.

The seasonality analysis showed only one month with statistically significant repeating AVM RSVs for the region worldwide. There was no seasonality detected for the AVM deaths per capita or USA AVM RSVs. Although seasonal patterns have been detected for other intracranial bleeding conditions, like subarachnoid hemorrhage, these results reflect that no prior research has detected seasonal patterns for AVM diagnosis or clinical presentation [29].

Regarding the quality of information analysis, there was not a suitable spread of websites among the categories; thus, a comparison is not appropriate. With regard to websites with and without the HONcode logo, it was not clear if there was better quality in either group. Websites with the logo showed higher ethical compliance given their superior scores in the HONcode and JAMA benchmark tools. This result was expected since the logo is only displayed on websites that the HON Foundation considers compliant with the foundation’s ethical principles. However, the JAMA benchmark scores were very low on average, as four websites achieved zero points, indicating a need for more widespread ethical compliance.

The DISCERN tool had four websites that qualified as “poor” and five that qualified as “good.” The average score for the tool was “good.” As DISCERN focuses primarily on educated choice of treatments, each of these websites has significant room for improvement in that domain. The CRAAP scores ranged from 15 to 30. As this tool focuses on overall information accuracy and authorship qualifications, we found there is a wide variance of scores, indicating an inconsistent level of reliability across the search. This uneven level of reliability may require patients to put forth unnecessary effort in order to find dependable sources of information.

The PEMAT understandability showed higher scores than the PEMAT actionability. This trend of superior understandability scores has been repeated across multiple studies regarding other health topics [20,30,31]. The average understandability score was 86.6%, while the average actionability score was 54.6%. Every website met the 70% cutoff for understandability, while only four met the cutoff for actionability. The average scores are considered understandable and poorly actionable. These results indicate a wide gap between the frequency of strategies for increasing understandability and those for increasing actionability. There is anecdotal evidence that information published more recently implements more strategies for actionability, but this study was unable to elucidate that trend given the limited sample size [20].

The readability analysis showed that no tool averaged at or below the recommended sixth-grade level, with only three of the total 54 scores calculated meeting that mark. Prior research has shown this trend of high reading levels required is common across many medical topics [32]. In fact, a systematic review analyzing 157 studies with 7891 total websites found that health information online in the United States and Canada is widely inappropriate for accurate use by the general population [33].

## Conclusions

As internet availability has become more widespread and plays a more integral role in patient self-education, an in-depth and broader understanding of online activity, information quality, and accessibility is imperative to optimizing this powerful tool. This GT analysis shows that online interest in AVMs as well as the related diagnoses of HHT and cerebral aneurysm is well correlated to the diagnosis quantity and, for AVM only, the attributed death quantity. This analysis indicates a relationship between interactions with healthcare regarding these diagnoses and subsequent patient-driven research. However, this study is limited in its ability to detect a causal relationship as well as by the limited data available.

Once patients access the internet, they need to safely rely on the information provided in an easily accessible format. Our study shows there is a wide variance in website ethics, treatment information quality, website/author qualifications, and actionable next steps. While some websites are strong in many facets, there is not a strong enough majority for patients to safely access complete information sources. While the quality of information was variable, the readability was uniformly too high of a grade level, a trend that is very common across healthcare information. Overall, this study showed that patients are routinely attempting to access information regarding these intracranial conditions in some relation to their contact with healthcare, but the information they obtain, specifically regarding AVMs, is not routinely reliable and the reading level required to understand is uniformly too high.
